# Analysis of the Influence of Thickness and Density on Acoustic Absorption of Materials Made from Used Cigarette Butts

**DOI:** 10.3390/ma14164524

**Published:** 2021-08-12

**Authors:** Valentín Gómez Escobar, Celia Moreno González, Guillermo Rey Gozalo

**Affiliations:** Research Institute for Sustainable Territorial Development (INTERRA), Department of Applied Physics, Polytechnic School, University of Extremadura, 10003 Cáceres, Spain; celiamg@unex.es (C.M.G.); guille@unex.es (G.R.G.)

**Keywords:** sound absorber, cigarette butts, sustainable material, recycling

## Abstract

The effects of the density and thickness of samples made from used cigarette butts on acoustic characteristics were analyzed in this study. All the analyzed samples showed high acoustic performance, indicating that the fabrication of acoustic absorbing material may be a good use for this problematic waste (due to its toxicity, continuous generation, lack of recycling method, etc.). An increase in either density or thickness shifted the absorption characteristics of the samples to lower frequencies and increased the overall absorption. The relationships of the frequency and value of the maximum absorption coefficient with thickness and/or density were analyzed. The shift of the maximum absorption coefficient value due to varying thickness is in good agreement with previous studies.

## 1. Introduction

Residual wastes are prevalent throughout the world, with cigarette butts being among the most important for several reasons.

Firstly, cigarette butts are present in almost all environments. Thus, they are usually found as a major element (in number and even in weight) in garbage [1,2,3]. The reason for this abundance is related to both the number of cigarettes consumed (5.7 trillion per year globally [4,5]) and the fact that large portions of used cigarettes are thrown to the ground [6], after which they are washed away by rain and river water to other sites.

Secondly, because filters (which are a part of used cigarette butts, with unsmoked tobacco) are mainly formed by a material (commonly cellulose acetate) that has a very low degradation rate, they persist in the environment for quite a long time (up to 10 years) [6,7].

Thirdly, during combustion, cigarette filters incorporate more than 130 chemical substances. These substances can leach into water, exposing different organisms to their toxic effects [8,9,10].

For the above reasons, as has been stated in a recent study by the World Health Organization [11], cigarette butts can be considered a serious environmental and public health problem.

Despite the previously mentioned problems associated with used cigarette butts, there are not many initiatives proposed for their selective collection and their recycling. Regarding the selective collection of cigarettes, they are generally collected together with the rest of the ordinary garbage. The use of containers for selective collection is not widespread, although there have been some proposals [12,13]. The initiatives for recycling were mostly summarized in two recently published papers [14,15]. Between them, the only application of this waste to construction materials is the inclusion of cigarette butts in construction bricks [16] and their utilization for developing acoustic absorbers, a research topic studied by the Lambda Acoustics Laboratory in the last several years [17,18,19,20]. Within this possible use as an acoustical absorber, materials made from used cigarette butts would have potential use for room conditioning, for noise barriers, or as a complement in solutions for acoustical isolation.

Several proposals have been made for the recycling of waste products other than cigarette butts into construction materials in general or acoustic absorbing materials in particular [21,22,23,24,25].

In previous studies related to the use of cigarette butts for making acoustic absorbing materials, the absorption of samples prepared with cigarette butts was quite satisfactory and found to be comparable to or even better than other materials conventionally used for absorption [17,18,19,20]. In the present study, we aimed to perform an in-depth investigation of the use of used cigarette butts for obtaining acoustic absorbing materials. Thus, an analysis of the influence of two important factors on acoustic absorption (the thickness and density of the samples) is presented. Understanding the effects of both factors is very important for guiding the design of future materials based on this waste.

## 2. Materials and Methods

### 2.1. Preparation of Samples

The samples used in the two studies described in this work had different origins.

Firstly, to investigate the influence of the compaction of the sample on the absorption coefficient, smoked cigarette butts were used. These used cigarette butts, which were from different brands, were retrieved from ashtrays or from the ground around buildings on the Campus of the University of Extremadura and its surroundings. They formed a very heterogeneous mixture; each butt had its original tobacco and blend of additives and different amounts of remaining unburnt tobacco. The remaining unsmoked tobacco was manually separated, and only the cigarette butts were taken. Before their use, cigarette butts were dried 24 h at 80 °C in order to eliminate their moisture content [26].

**Figure 1:**
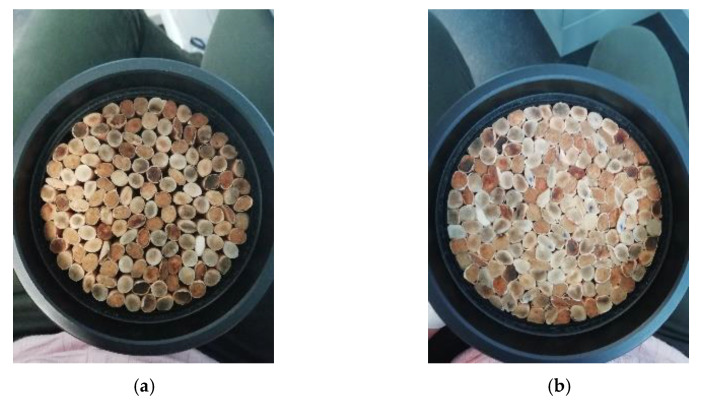
Example pictures of some of the prepared samples for the compaction study. Samples prepared with 140 (**a**) and 180 (**b**) used cigarette butts.

For the compaction study, butts were separated by length in order to minimize their inhomogeneity. Samples were prepared by putting 10–15 butts in the 29 mm holder or 130–160 in the 100 mm holder. Table 1 summarizes the main characteristics of the groups of cigarette butts used for the different samples (average length and diameter of the filters), as well as the range of butts used and the range of density of the samples prepared for this study. Figure 1 presents two pictures of samples prepared for the 100 mm holder, with different numbers of used cigarette butts (140 and 180).

**Figure 2:**
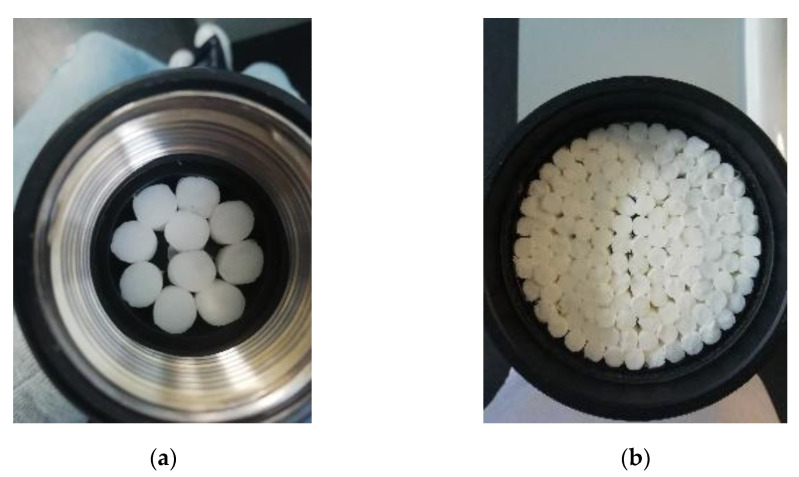
Example pictures of some of the prepared samples for the thickness study in the 29 mm holder (**a**) and 100 mm holder (**b**).

Secondly, to study the influence of the length of the filters on the absorption characteristics of the samples (influence of the thickness of the sample), unused filters with a length of 85 mm were provided by a distributor of filters (final filters for cigarettes were obtained by cutting these 85 mm filters). For this study, these filters were manually cut into the following lengths: 9.5, 19, 28, 38, 57, 67, 75, and 85 mm. Samples made with these cut filters included 10 filters for the 29 mm diameter holder (Figure 2a) and 140 for the 100 mm diameter holder (Figure 2b).

### 2.2. Instrumentation for Acoustic Absorption Determination

**Figure 3:**
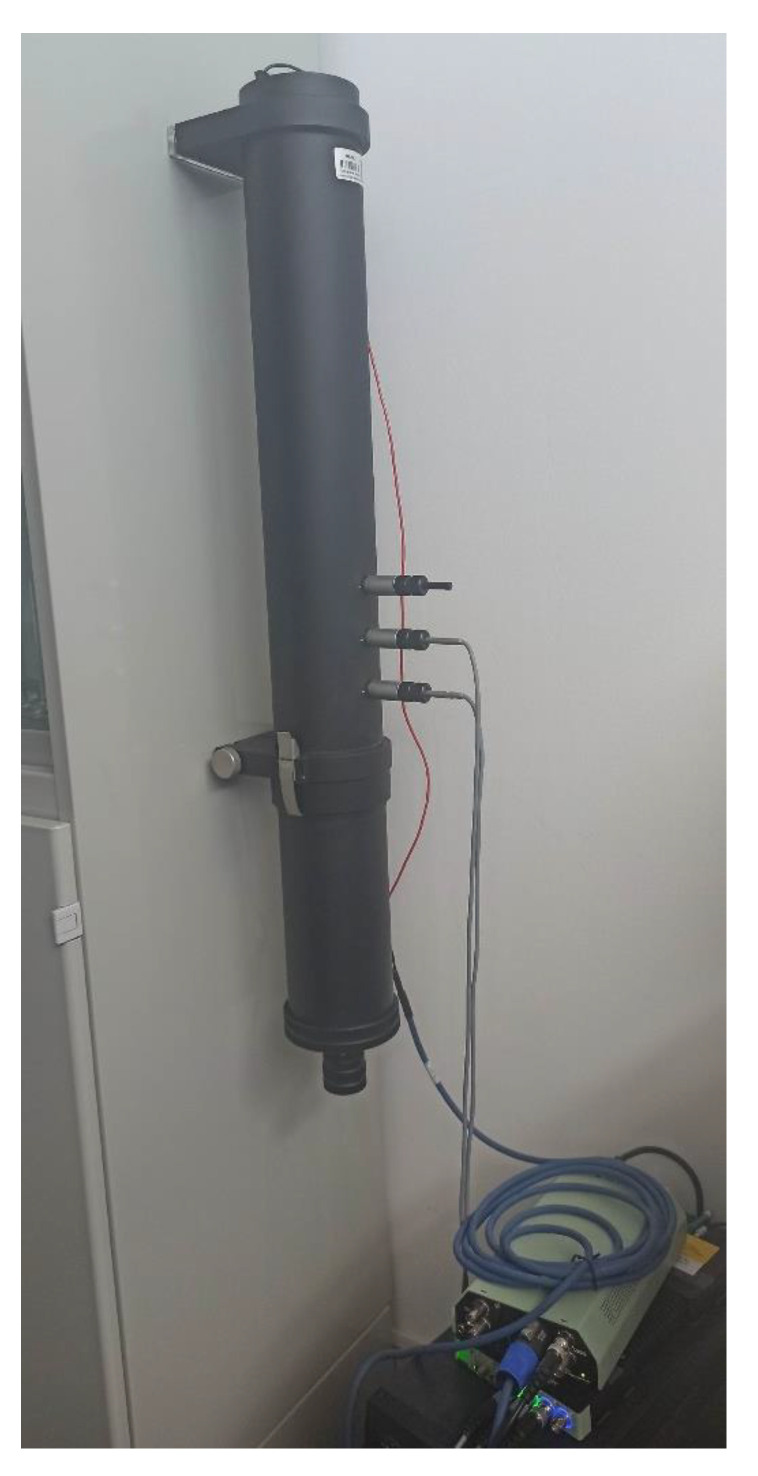
Impedance tube disposition used for the measurements: 100 mm holder.

The absorption coefficients for different samples were measured using the Impedance Tube Kit (Type 4206,Hottinger Brüel & Kjaer Ibérica, Nærum, Denmark), equipped with two quarter-inch condenser microphones (Type 4187, Hottinger Brüel & Kjaer Ibérica, Nærum, Denmark). As the prepared samples could be considered nonconsolidated, the tube was placed in a vertical position (Figure 3). The signals were analyzed using a portable Brüel and Kjær PULSE System with four input data channels (Type 3560-C, Nærum, Denmark). Two sample holders with diameters of 29 mm (valid in the frequency range of 500 Hz to 6400 Hz) and 100 mm (valid in the frequency range of 50 Hz to 1600 Hz) were used.

The sound absorption coefficients of different samples were determined using an impedance tube following the two-microphone transfer function method described in the ISO 10534-2 standard [27].

## 3. Results and Discussion

### 3.1. Analysis of the Influence of Compaction of the Samples on Their Acoustic Behavior

As mentioned previously, the used cigarette butts in this study could be grouped into three different lengths (approximately 14, 21, and 26.5 mm). As discussed further, the length of the butts (and, thus, the thickness of the sample) has an important influence on the absorption spectra of the samples. Thus, for the present study, almost all results are presented independently for each butt length.

**Figure 4:**
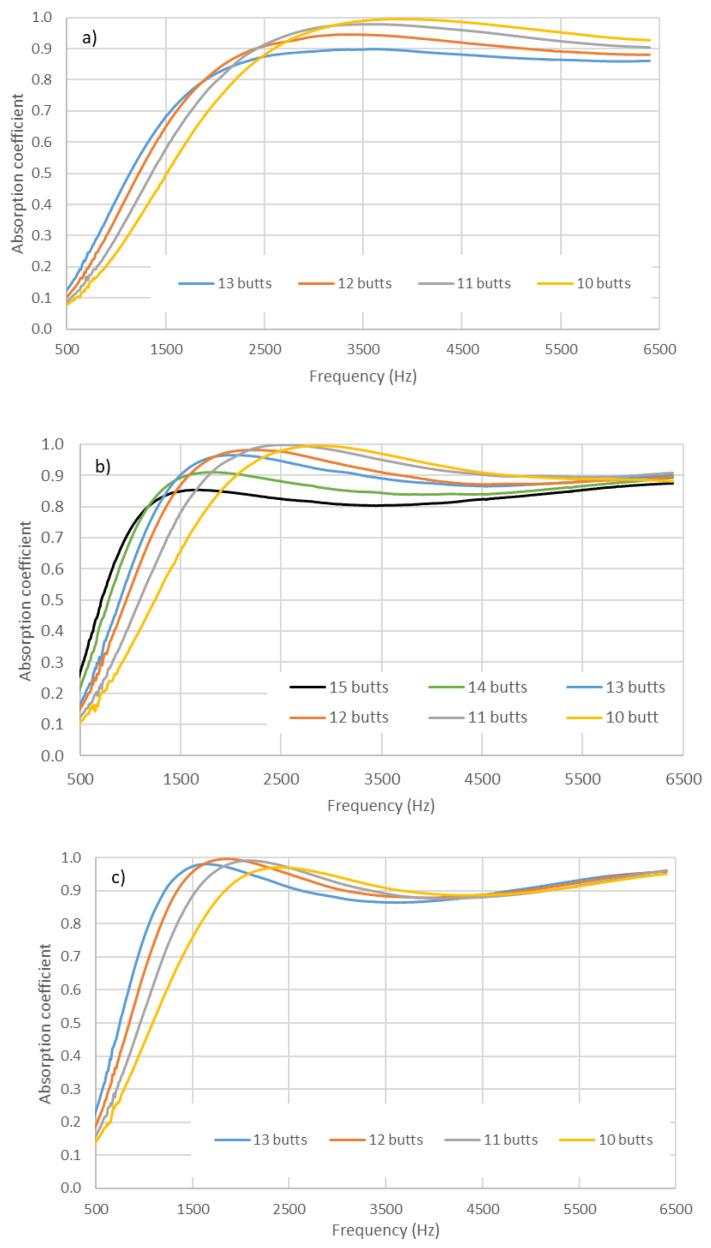
Variation in absorption coefficient with compaction (number of used butts) in the holder (29 mm). Length of butts: (**a**) 14.2 ± 0.8 mm; (**b**) 20.8 ± 0.4 mm; (**c**) 26.6 ± 0.4 mm.

**Figure 5:**
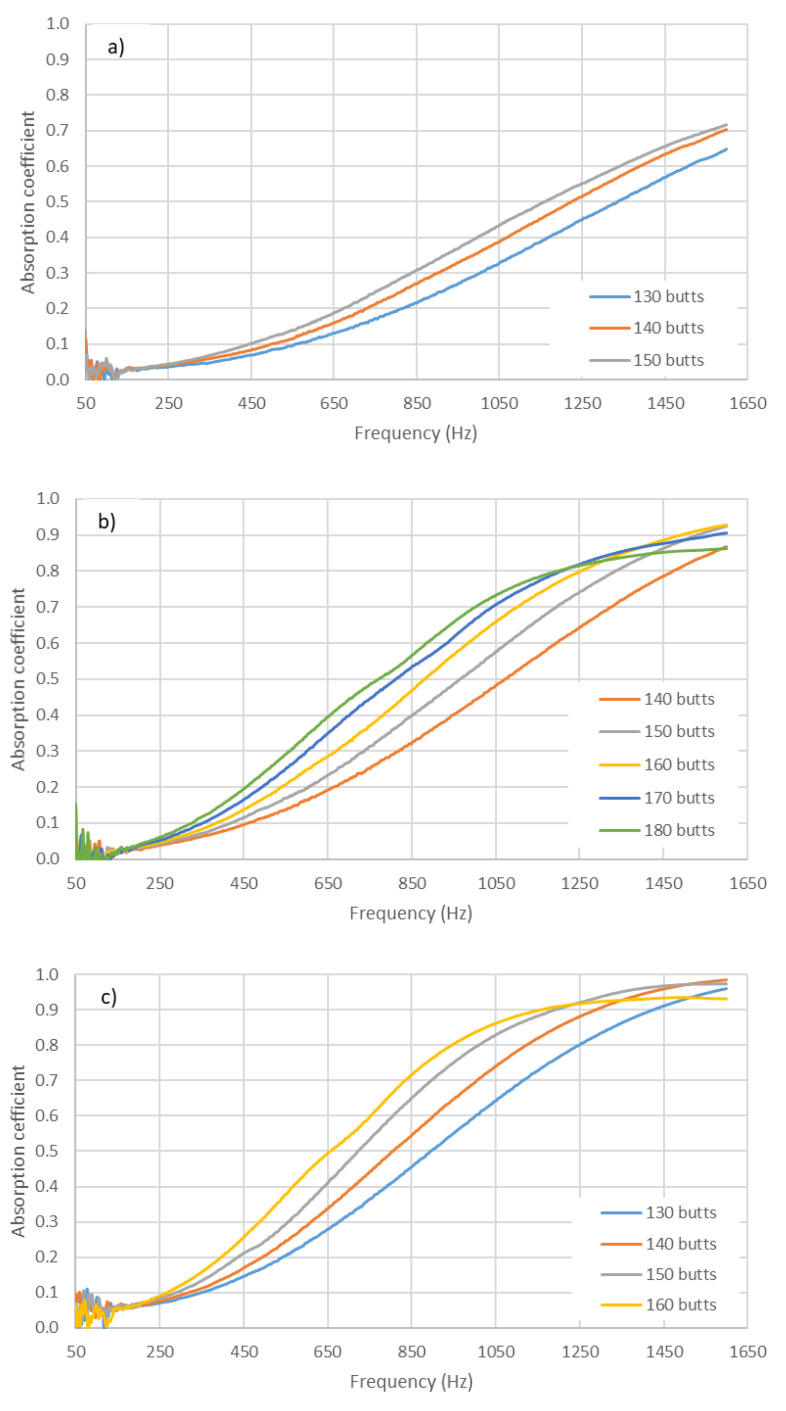
Variation in absorption coefficient with compaction (number of used butts) in the holder (100 mm). Length of butts: (**a**) 14.3 ± 0.8 mm; (**b**) 20.8 ± 0.2 mm; (**c**) 26.4 ± 0.7 mm.

In Figure 4 and Figure 5, the average absorption coefficients measured for the prepared samples are shown for the 29 mm and 100 mm impedance tube holders, respectively.

**Figure 6:**
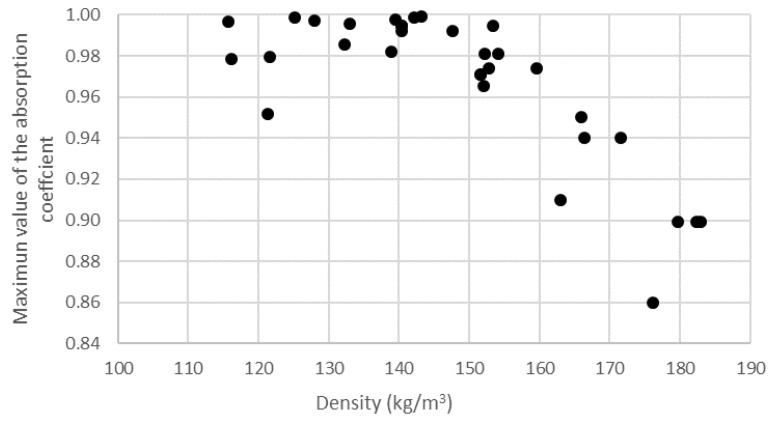
Variation in the value of the maximum absorption coefficient with compaction (density) in the 29 mm holder.

In Figure 4 and Figure 5, firstly, comparing the different graphs within each figure reveals that the values shifted to lower frequencies as the length of the butts increased; this is discussed in the next section when analyzing the influence of the thickness of the sample on the acoustic behavior. Secondly, it can also be observed that, in each figure, the increase in the number of butts used in the preparation of the sample also produced a shift to lower frequency values. The shift in the value of the maximum frequency to lower frequencies when increasing the density was seemingly accompanied by a decrease in the value of the maximum absorption coefficient. Thus, in Figure 6, it can be observed that the value of absorption tended to decrease as density increased.

**Figure 7:**
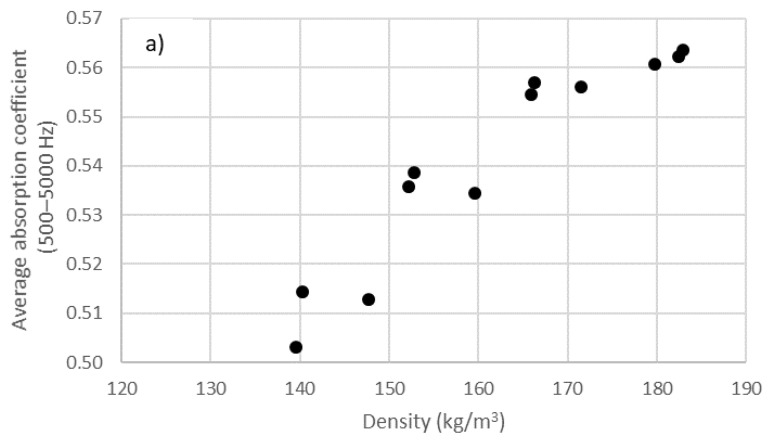

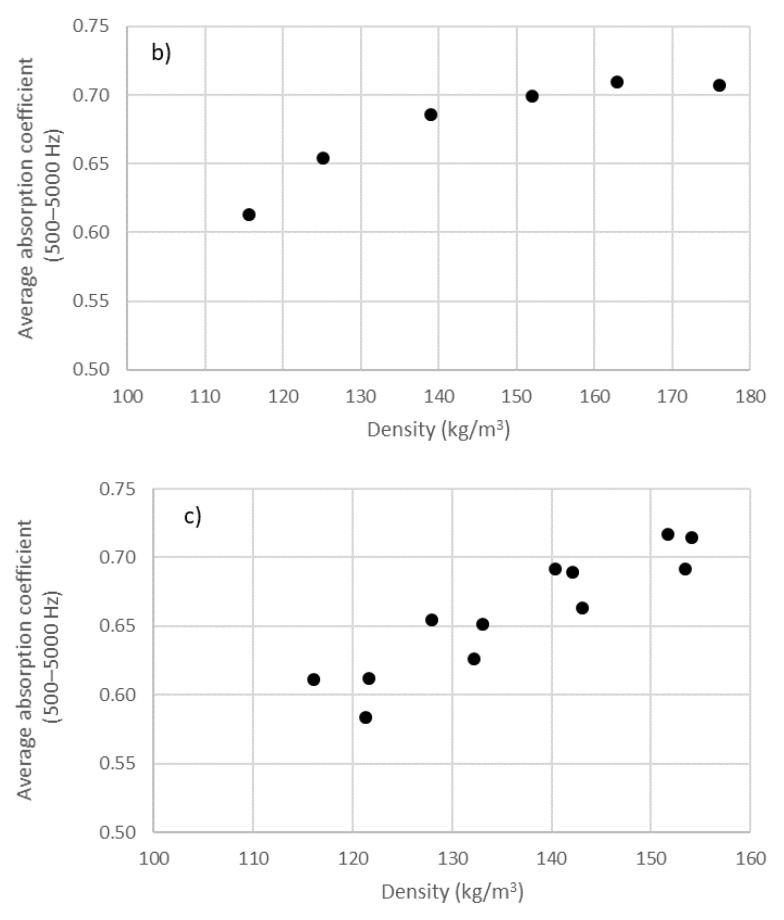
Variation in average absorption coefficient in the range of 500–5000 Hz octave bands with compaction (density) in the 29 mm holder: (**a**) 14.2 ± 0.8 mm; (**b**) 20.8 ± 0.4 mm; (**c**) 26.6 ± 0.4 mm.

Although, from this last figure, one might infer that sample absorption decreased as the density increased, in the density range studied, the opposite was the case. Thus, in Figure 7, an increase in the overall absorption of the sample (in the validity range of the 29 mm diameter holder) with increasing density was observed for the three lengths of butts, and there was a clear increase in absorption with increasing density. Thus, in the studied range, an increase in density produced an increase in the overall absorption but a decrease in the value of the maximum absorption.

### 3.2. Influence of the Thickness of the Samples

As mentioned previously, eight different lengths of cigarette filters were used for the present study. For each filter length, six different samples were prepared for the 29 mm diameter holder, and three different samples were prepared for the 100 mm diameter holder.

**Figure 8:**
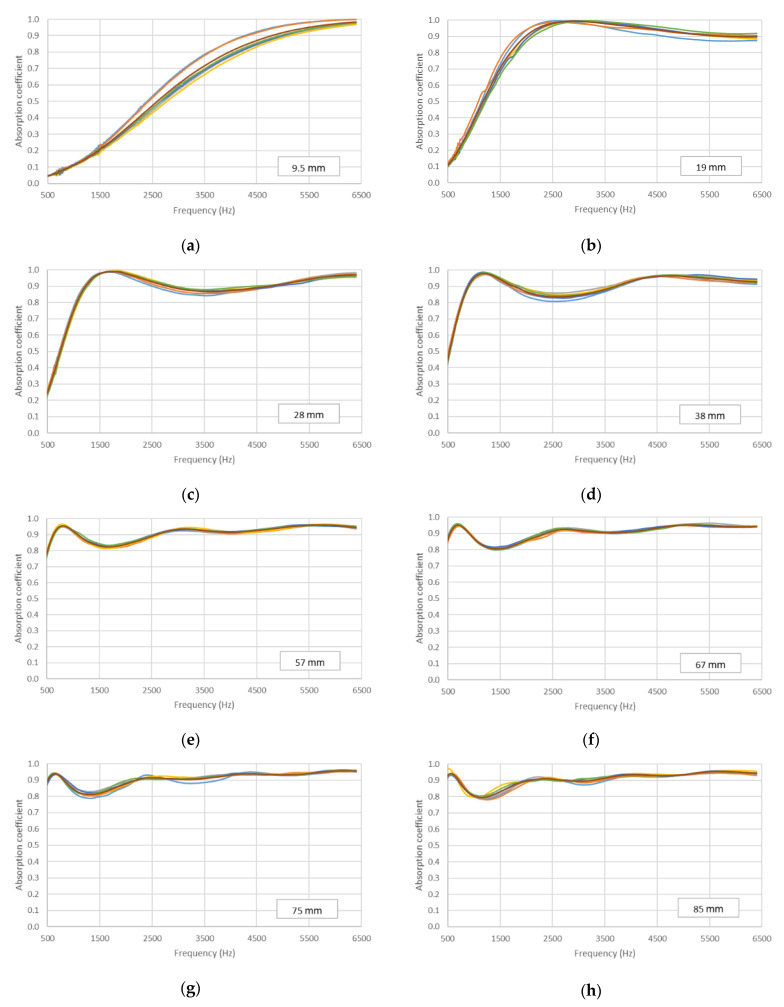
Variation in absorption coefficient with the length of the filter for the six samples (each one with different color) studied: 29 mm diameter holder. (**a**) 9.5 mm, (**b**) 19 mm, (**c**) 28 mm, (**d**) 38 mm, (**e**) 57 mm, (**f**) 67 mm, (**g**) 75 mm, (**h**) 85 mm.

**Figure 9:**
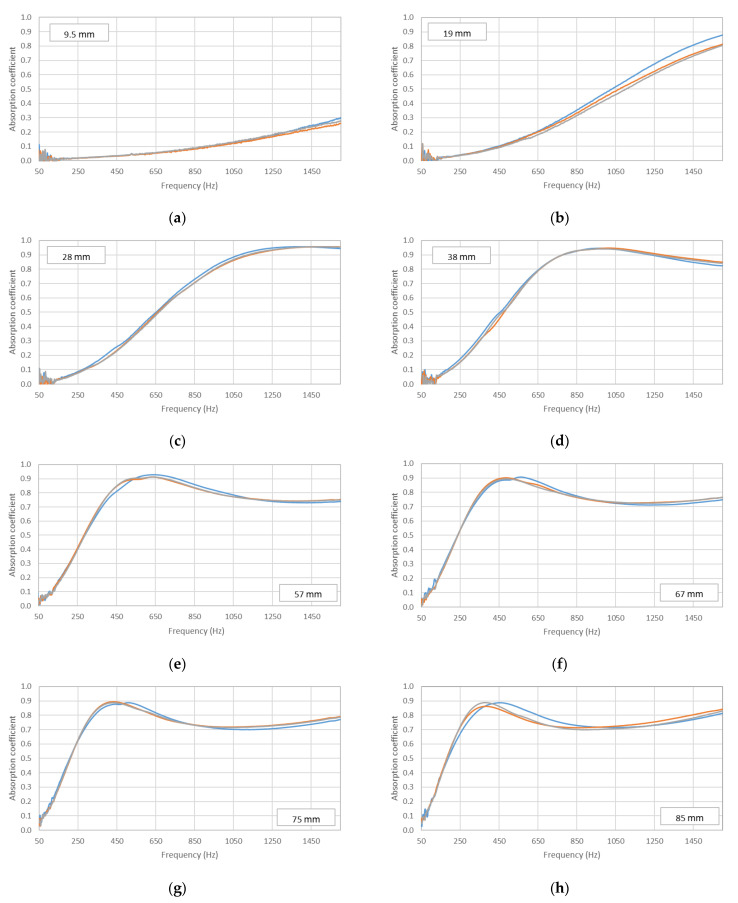
Variation in absorption coefficient with the length of the filter for the three samples (each one with different color) studied: 100 mm diameter holder. (**a**) 9.5 mm, (**b**) 19 mm, (**c**) 28 mm, (**d**) 38 mm, (**e**) 57 mm, (**f**) 67 mm, (**g**) 75 mm, (**h**) 85 mm.

The results obtained for the different samples of each filter length were compared for the two different size holders and can be observed in Figure 8 and Figure 9. For the 29 mm diameter holder, the results in Figure 8 show a clear similarity in the acoustic behavior of the six samples for each filter length. This similarity can also be observed for the 100 mm holder (Figure 9); in this case, the results are presented for the three samples of each filter length made for this holder size.

**Figure 10:**
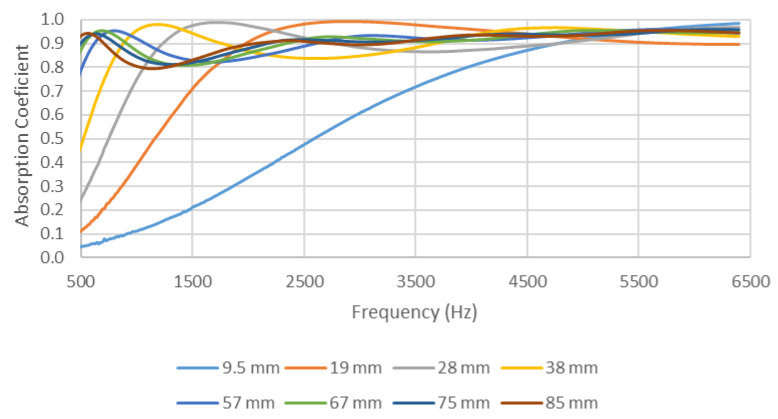
Variation in absorption coefficient with the length of the filter: 29 mm diameter holder.

**Figure 11:**
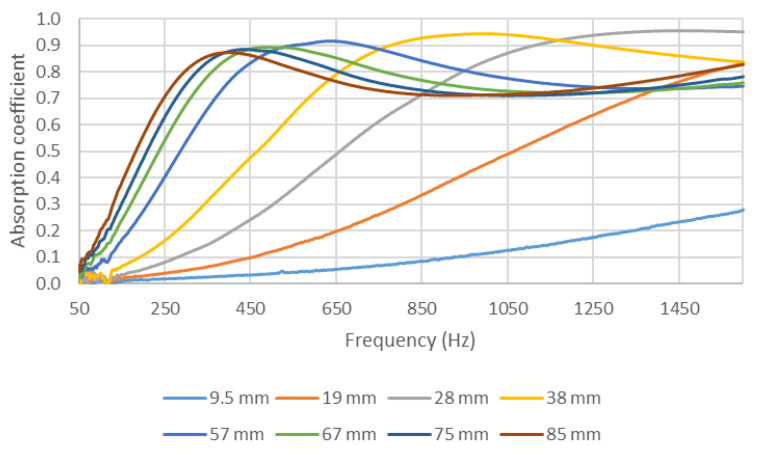
Variation in absorption coefficient with the length of the filter: 100 mm diameter holder.

The homogeneity of the absorption values obtained in the different samples for the same length was analyzed through dispersion parameters. Thus, the coefficient of variation (CV) was used to analyze the representativeness of the average value. A small CV indicates that the values were concentrated around the average and, therefore, there was little variability, and the average value was representative. Although there are no universal criteria, values below 20% are usually considered low [28]. The highest coefficients of variation are shown in Figure 8 for the 9.5 mm and 19 mm lengths in some frequency ranges. However, these values did not exceed 15%. The coefficients of variation for the remaining lengths did not exceed 5%, and, for most frequencies, the CV values were between 1% and 2%. Therefore, from this analysis, it can be concluded that the average value was representative of the samples analyzed with the 29 mm diameter holder. Figure 9 also shows low coefficients of variation (<10%) except in the frequency range from 50 to 150 Hz, in which some coefficients of variation were higher than 20%. In any case, the average was sufficiently representative of the values obtained in the different samples measured with the 100 mm diameter holder. Having confirmed the representativity of the average value, the average absorption values of the six samples made for each filter length are shown in Figure 10 and Figure 11 for the two diameter holders.

Comparing the results shown in Figure 10 and Figure 11 reveals a shift of the first absorption maximum to lower frequencies when increasing the length of the filter used (and, thus, the thickness of the sample). This was due to the fact that, when increasing the thickness of the sample, the sample acquired a new maximum speed for waves in the air particulate, thus improving its acoustic behavior at lower frequencies.

**Figure 12:**
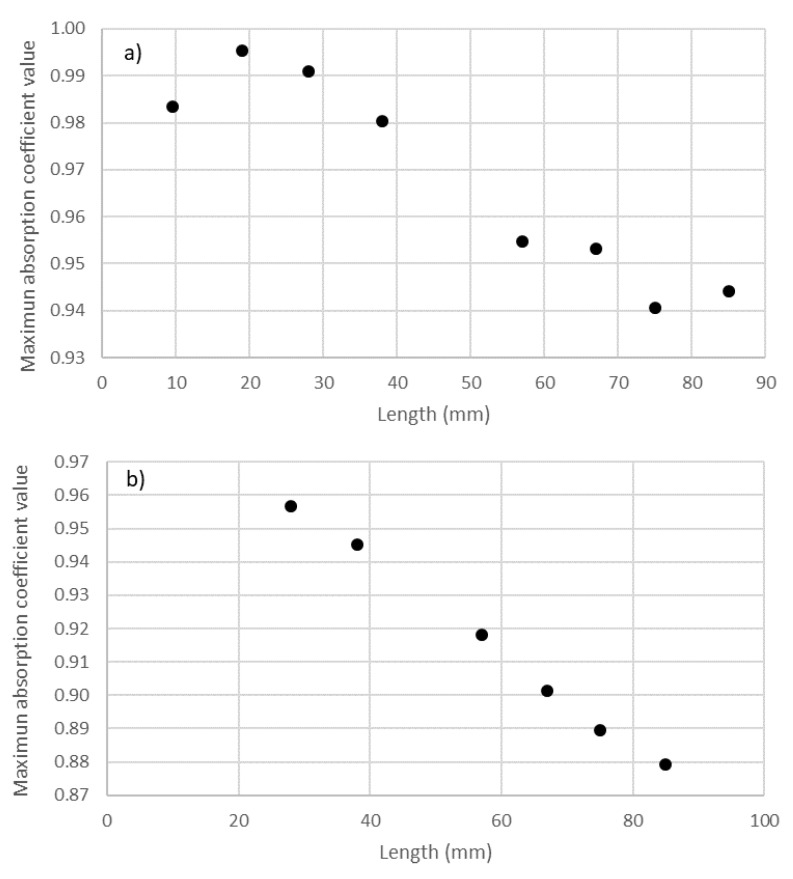
Variation in the value of the maximum absorption coefficient with thickness: (**a**) 29 mm holder; (**b**) 100 mm holder.

**Figure 13:**
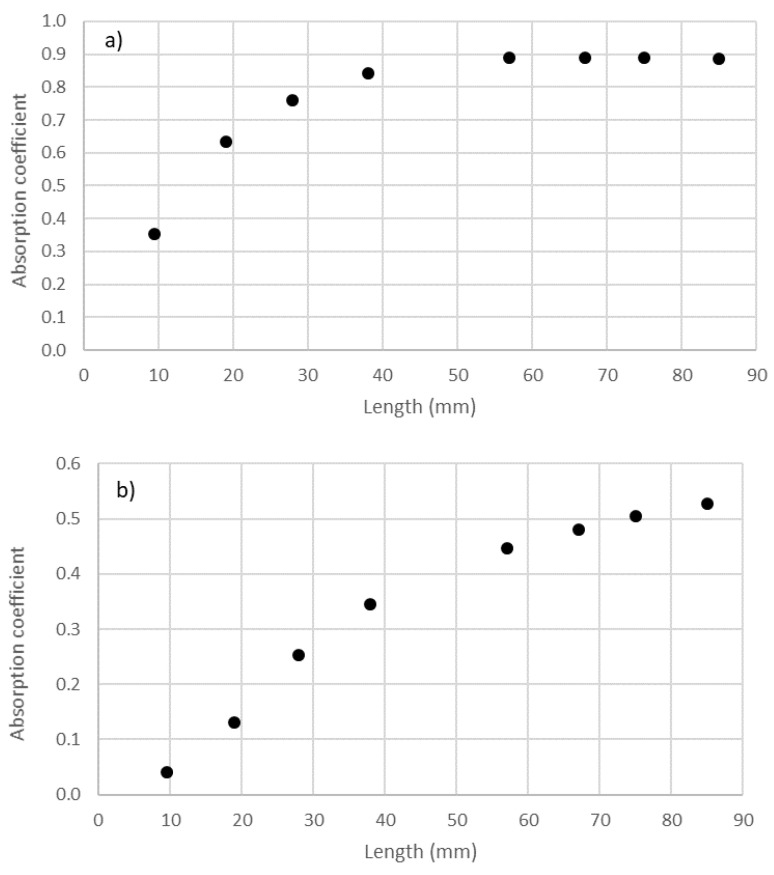
Variation in average absorption coefficient with the length of cigarette butts (thickness): (**a**) range of 500–5000 Hz octave bands for the 29 mm holder; (**b**) range of 50–1250 Hz octave bands for the 100 mm holder.

As in the study of the variation in the absorption of the samples with density, it can also be observed that the shift in the graphs to lower frequencies when increasing thickness also produced a decrease in the maximum value of the absorption coefficient. This can be observed in Figure 12 for the two holders. Similar to the previous study of the influence of density, this reduction in the maximum value of absorption did not imply a variation in the overall absorption of the sample. The overall absorptions in the validity ranges of the 29 mm holder (octaves from 500 to 5000 Hz) and of the 100 mm holder (octaves from 50 to 1250 Hz) are shown in Figure 13.

In Figure 12a, it is important to note that the value with a filter length of 9.5 mm behaved differently from those with other lengths, presumably because the maximum was lower than 500 Hz and, thus, out of the studied range of frequency. As the maximum was not in the studied range for the 100 mm holder, data for the lengths of 9.5 and 19.5 mm were not included in Figure 12b.

Furthermore, in Figure 13, it is important to note that, in the range of the length of the filters studied, at lower values, the increase in the length (and, thus, as mentioned, in the thickness) implied an increase in the overall absorption in the two octave ranges (500–5000 Hz and 50–1250 Hz for the 29 mm and 100 mm holder, respectively). However, for the 29 mm holder results, this increase stopped near 50 mm of thickness, after which the overall absorption remained constant. This result was similar to the saturation behavior for the samples (acoustic saturation).

Lastly, analyzing the value of the maximum absorption frequency in Figure 10 reveals that the value clearly shifted when increasing the thickness of the sample. This shift of the maximum absorption frequency with the increasing thickness of the samples has also been described in previous studies and compares well with the shift in other samples (glass wool) showing linear behavior [19,21].

**Figure 14:**
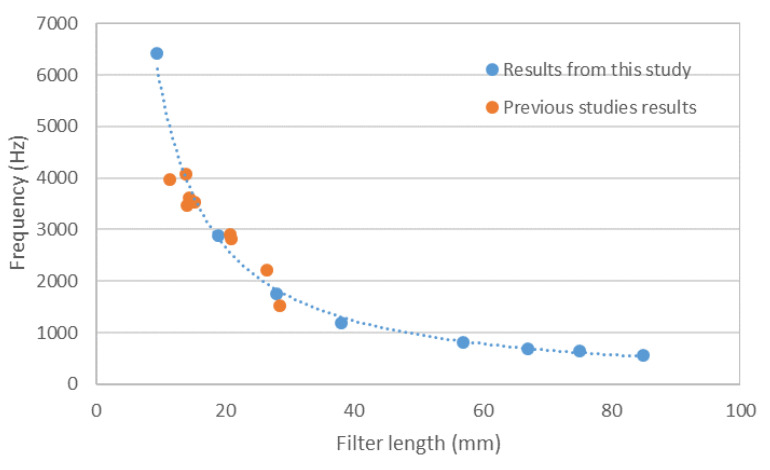
Comparison of the variation in frequency of the maximum absorption coefficient with the length of the filters of the samples between this study and previous ones.

Representing the values of the maximum absorption frequency values with the size of the filter (and, thus, the thickness of the sample), it can be seen (Figure 14) that the behavior was far from linear.

The reason for the difference between this study and previous research is the range of thicknesses of the samples (from 10 mm to 30 mm in previous studies; from 9.5 to 85 mm in this one). Indeed, overlapping the results obtained with used cigarette butts in the previous studies [19,21] with those of the present study, it can be seen, in the same figure, that the results of all three works are coherent.

## 4. Conclusions

In this work, the effects of the density and thickness of samples on the acoustic behavior of the material (used cigarette butts) were analyzed.

In the studied range, an increase in density improved the absorption of the samples at medium frequencies. The frequency of the maximum absorption coefficient shifted to lower frequencies when increasing the density, although the value of the maximum absorption coefficient tended to decrease. Observing the overall absorption in the 500–5000 Hz octave bands revealed that the absorption increased with increasing density in the studied range (from 110 to 160 kg/m^3^). It is important to note that, for all densities analyzed, the absorption coefficients were higher than 0.8 for frequencies over 2000 Hz, corroborating the good acoustic behavior of samples prepared with used cigarette butts observed in previous studies [15,16,17,18].

In the study of the thickness of the samples, the overall absorption in the 500–5000 Hz octave bands increased when increasing the thickness until reaching a state similar to saturation at a thickness of approximately 5 mm. As with density, an increase in thickness improved the absorption of samples at low and medium frequencies. As thickness increased, the frequency of the maximum absorption coefficient shifted to lower frequencies, and the maximum absorption coefficient value decreased. Analyzing this change in the frequency of the maximum, it can be concluded that the linearity observed between the value of this frequency and the thickness of the sample was restricted by the low range of thicknesses studied. In the studied range in this work, the relationship between the values of the maximum absorption frequency and the thickness was clearly nonlinear. The previous study results are consistent with the behavior observed in this study. Values of thickness higher than 57 mm led to absorption coefficients over 0.8 for frequencies over 500 Hz, indicating the potential of materials made with cigarette butts as acoustic absorbers with absorption coefficients similar to or even higher than other commercial absorbers [15,16].

Further studies can emerge following the results of this work. For instance, it could be interesting to analyze the influence of the occupied surface of the sample (as can be seen in Figure 1 and Figure 2, the entire surface of the holders is not occupied by the cigarette filters) in the acoustical behavior of samples. Furthermore, the study of other sample configurations (for instance, disaggregating the cigarette butts or using perforated panels) may be more useful in building engineering or in the development of noise barriers.

## Figures and Tables

**Table 1:** Summary of characteristics of samples used for the compaction study (all smoked filters).

**Sample Number (29 mm Holder)**	**Average** **Length (mm)**	**Average Diameter (mm)**	**Range of Butts**	**Range of Density** **(kg/m^3^)**
12	14.2 ± 0.8	8.04 ± 0.08	10–13	141–186
6	20.8 ± 0.4	7.86 ± 0.12	10–15	116–176
12	26.6 ± 0.4	7.88 ± 0.13	10–13	117–154
**Sample Number (100 mm holder)**	**Average** **Length (mm)**	**Average Diameter (mm)**	**Range of Butts**	**Range of Density** **(kg/m^3^)**
9	14.3 ± 0.8	8.00 ± 0.12	130–150	156–182
5	20.8 ± 0.2	7.88 ± 0.05	140–180	138–179
7	26.4 ± 0.7	7.83 ± 0.18	130–160	132–163

## Data Availability

The data presented in this study are available on request from the corresponding author.
